# [Retracted] Knockdown of human antigen R reduces the growth and invasion of breast cancer cells *in vitro* and affects expression of cyclin D1 and MMP-9

**DOI:** 10.3892/or.2026.9078

**Published:** 2026-02-16

**Authors:** Zhu Yuan, Andrew J. Sanders, Lin Ye, Yu Wang, Wen G. Jiang

Oncol Rep 26: 237–245, 2011; DOI: 10.3892/or.2011.1271

Following the publication of the above paper, a concerned reader drew to the Editor's attention that, for the RT-qPCR experiments shown in Fig. 2A on p. 240, data which were intended to show the results of experiments performed in two different cell lines (MCF7 and MDA-MD-231) appeared to have been duplicated, with some evidence of stretching of the bands. In addition, it was shown that, in Fig. 5A and C on p. 242, data which were also intended to show the results of RT-qPCR and western blot analysis experiments performed in the same two cell lines appeared to have been duplicated; moreover, GAPDH data in Fig. 2C also appeared to have been included in Figs. 5C and 6C, but with the data bands shown in different orientations, and probable rearrangement of bands had occurred with the cyclin D1 / MMP-9 data in Fig. 5C compared with Fig. 6C.

Owing to the large number of duplications of data that have been identified in this paper, the Editor of *Oncology Reports* has decided that it should be retracted from the Journal on account of a lack of confidence in the presented data. The authors were asked for an explanation to account for these concerns, but the Editorial Office did not receive a reply. The Editor apologizes to the readership for any inconvenience caused.

